# Supplementary material for: Combination of Radiomics and Machine Learning with Diffusion-Weighted MR Imaging for Clinical Outcome Prognostication in Cervical Cancer
Source: Tomography. 2021 Aug 5;7(3):344–57. doi: 10.3390/tomography7030031 (PMC8396356; doi:10.3390/tomography7030031)
Supplement: Supplementary file 1 [file tomography-07-00031-s001.zip › tomography-1284741-supplementary.pdf]

Supplementary Materials

1. Data Availability

The code is made available publically at https://github.com/enwrap/Cervix.

Table S1. Using Pearson coefficient method of cut-off, the final set of features has 24 radiomics features listed are: two first-order features; two GLDM gldm features; two GLSZM features and 18 wavelet features ((i) Six first-order features; (ii) two GLDM features; (iii) seven GLSZM features; (iv) one GLCM feature; (v) two GLRLM feature).

Radiomics Features	Minimum Value	Maximum Value	Mean Value
original_gldm_SmallDependenceLowGrayLevelEmphasis	0.000552	0.007343	0.001823
original_glszm_LowGrayLevelZoneEmphasis	0.002798	0.028934	0.008349
original_glszm_SmallAreaLowGrayLevelEmphasis	0.001902	0.020863	0.005429
wavelet_HHH_glcm_ClusterShade	−0.40966	0.185952	−0.00789
wavelet_LLL_gldm_DependenceEntropy	7.356461	8.249372	7.829342
wavelet_LLL_firstorder_Uniformity	0.016999	0.043945	0.031423
wavelet_LLL_glrlm_GrayLevelNonUniformityNormalized	0.016802	0.042434	0.030712
wavelet_HLL_glszm_GrayLevelVariance	1.164063	42.63017	19.15147
wavelet_HLH_gldm_SmallDependenceHighGrayLevelEmphasis	0.230895	229.3142	95.05165
wavelet_HLH_firstorder_Median	−1.24135	0.634306	−0.05558
wavelet_HLH_glszm_GrayLevelVariance	1.1195	30.01649	17.17258
wavelet_HLH_glszm_SmallAreaHighGrayLevelEmphasis	4.752931	707.1599	291.0122
wavelet_HHH_glszm_ZoneEntropy	3.883709	5.103177	4.430195
wavelet_HHL_glrlm_RunEntropy	2.757067	3.673218	3.303805
wavelet_HHL_glszm_GrayLevelVariance	0.25856	12.1992	5.714234
wavelet_HHL_glszm_ZoneEntropy	4.111524	5.615251	4.7727
original_firstorder_Energy	6.66E + 08	3.83E + 10	8.67E + 09
original_firstorder_TotalEnergy	8.22E + 08	7.87E + 10	1.29E + 10
wavelet_LHH_firstorder_TotalEnergy	6399921	5.06E + 08	1.33E + 08
wavelet_HHH_glszm_LargeAreaHighGrayLevelEmphasis	304599.1	54370308	9604343
wavelet_HHL_firstorder_TotalEnergy	529500	2.68E + 08	55719105
wavelet_LLL_firstorder_Energy	5.32E + 09	3.02E + 11	6.83E + 10
wavelet_LLL_firstorder_TotalEnergy	6.6E + 09	6.26E + 11	1.02E + 11
original_gldm_DependenceEntropy	6.684414	7.504257	7.024256

Table S2. The baseline and follow up ADC values in the various groups with percentage change in ADC. There is an obvious change in ADC in the recurrence and metastatic groups. The nodal positive and negative groups did not show much of change.

	Recurrence	No Recurrence	Lymph Node	Node Negative	Metastasis	Non-Metastatic
Baseline ADC	0.856	0.893	0.890	0.867	0.921	0.795
Follow up ADC	1.519	1.435	1.447	1.477	1.459	1.441
Percentage change in ADC	77.4%	43.5%	44.7%	47.7%	77.4%	44.1%

2. Methods

The patients included in the study were fulfilling the following criteria. The inclusion criteria were: histopathological diagnosis of squamous cell carcinoma of uterine cervix, non-pregnant/not breastfeeding female patients with no previous gynecological malignancies, and no contraindications for MRI. All patients who received definitive radiation treatment with curative intent, as determined at the initial consultation, were included. Out of these 31 patients faced exclusion from the study due to the following reasons: primary cervical lesions were not detectable on MRI (n = 1), lesion boundaries not succinctly discernible on diffusion images or due to poor spatial resolution (n = 8), poor quality of the diffusion images (n = 3), whose tumor contained only one slice (n = 7), patients who have contraindications to enter magnetic field like those with a MR incompatible pacemaker, cochlear implant or any other metallic prosthesis that interferes with the examination (n = 2) and patients who refuse or were lost to follow up (n = 9), and whose tumor histological type was not squamous cell carcinoma (n = 1).

Details of Extracted Radiomic Features:

These 851 radiomic features include:

1. Eighteen first-order features: Commonly used and basic metrics are used to describe the voxel distribution intensities within the image region defined by ROI.
2. 14 shape features: These features describe the shape and size of the region defined by the mask.
3. Fourteen gldm features: A Gray Level Dependence Matrix (GLDM) quantifies gray level dependencies, defined as the numbers of neighboring voxels from the center voxel, neighboring voxels are within distance δ from the center voxel.
4. Twenty-two glcm features: A Gray Level Co-occurrence Matrix (GLCM) expresses the combinations of discretized intensities (gray levels) of neighboring voxels in a 3D volume.
5. Sixteen glrlm features: A Gray Level Run Length Matrix (GLRLM) quantifies gray level runs, which are defined as the number of consecutive and collinear points with the same gray level value in the run.
6. Sixteen glszm features: A Gray Level Size Zone counts the number of connected voxels with the same gray level intensity. Voxels are connected if the Chebyshev distance is 1. $\delta = \max_i |x_i - y_i|$
7. Five ngtdm features: The neighborhood gray-tone difference matrix (NGTDM) is defined as the sum of gray level differences between the average discretized gray level of neighboring voxels and voxels with discretized gray level. The voxels are considered neighboring voxels if they are within a Chebyshev distance δ.
8. Seven hundred forty-four wavelet features: Wavelet features generated eight decompositions per level (High or Low pass filter are applied in all possible combinations in each of the dimensions of a 3-D image).

Details of Statistical Analysis

AUC, "Area under the ROC Curve" ranges in value from 0 to 1. AUC is 0 when false negatives and false positives cover 100% of the data, and AUC is 1 when there are no false negatives and no false positives. AUC measures the ranking of good predictions and ensures the class prediction variable with different threshold values. But AUC has some limitations, and thus, Cohen's Kappa is also considered another parameter to compare the various models that measure the agreement between two raters. It is more robust than a simple percent agreement calculation. $\{K = (Po - Pe)/(1 - Pe) = 1 - ((1 - Po)/(1 - Pe))\}$. Cohen's Kappa and AUC were used to compare the prediction capability of various features using different classification algorithms for clinical prognostication.

Details of Interpretation from Heat Map

Seven radiomics features: (i) wavelet HHH GLSZM Large Area High Gray Level Emphasis (LAHGLE), (ii) wavelet HHL first order Total Energy (TE), (iii) wavelet LHH first order Total Energy (TE), (iv) original first order Energy, (v) original first order Total Energy, (vi) wavelet LLL first order Energy, and (vii) wavelet LLL first order Total Energy are the features which generally have value higher than the mean value in the cases in which Lymph Node and metastasis are present. At the same time, 3 radiomics features: (i) original gldm Small Dependence Low Gray Level Emphasis (SDLGLE), (ii) original GLSZM Small Area Low Gray Level Zone Emphasis (SALGZE), and (iii) original GLSZM Low Gray Level Zone Emphasis (LGLZE) possess slightly higher value than the mean value in the patients in which Lymph Node is absent. LGLZE possess slightly higher value than the mean value in the patients in which Metastasis is present Thus, we propose the following hypothesis: If the value of 7 features is greater than mean value then there are high chances of lymph nodes or metastasis to be present and if the value of the 3 features is more than the average value then the probability of absence of lymph nodes or metastasis is high.

The figure III shows the correlation between different radiomics and ADC features with the two classes of Recurrence (Absent and Present). 17 radiomics features shown on the right side might have value higher than the average to conclude the presence of recurrence or 4 radiomics features: (i) wavelet HLH first order Median, (ii) original gldm SDLGLE, (iii) original GLSZM SALGZE, and (iv) original GLSZM LGLZE posses slightly higher value than the mean value in the patients in which Recurrence is present. Thus, a hypothesis can be generated as follows: If the value of 17 features is greater than mean value then there are high chances of recurrence to be present and if the value of the 3 features is more than the average value then the probability of presence of recurrence is high.

The correlation between different radiomics and ADC features with the 4 classes of Stage (Stage I, II, III, and IV). Stage II and III shows high similarity between the radiomics features trends. It is difficult to generate a hypothesis because of highly skewed data with a small sample size. The heat map generated from analysis of the

same is depicted in supplementary files. The figure IV shows correlation of each feature with clinical outcomes desired in this study design depicted in the heat map. It is observed that each radiomics feature has a correlation value greater than 0.3 with at least one of the clinical outcome whereas ADC parameters do not share the same degree of correlation. The details of multiple classifiers and algorithms with the corresponding AUC and kappa values for the sought clinical outcomes are mentioned in a tabulated sheet (Tables 2–5). Since this study was an effort to find the best classifier among the multitude of machine learning algorithms, a line plot depicting the AUC and kappa for each relevant classifier was created for all relevant clinical outcomes as depicted in supplementary files.

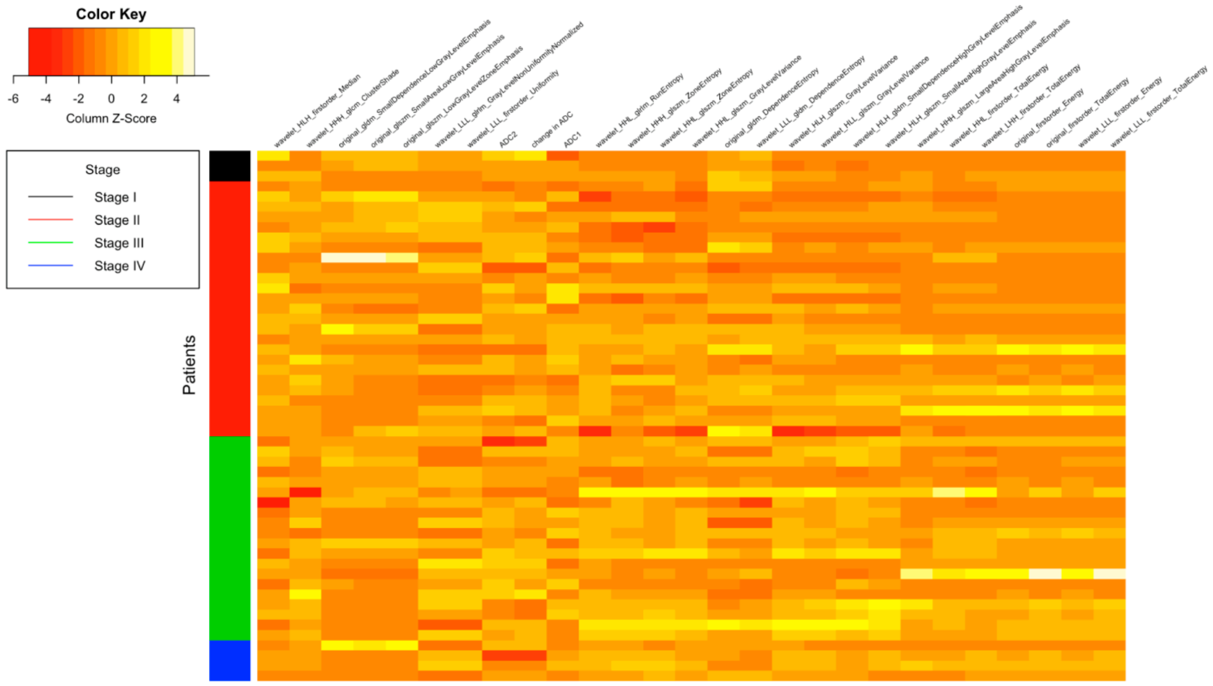

Figure S1. Heat map showing correlation between different radiomics and ADC features with Stages.

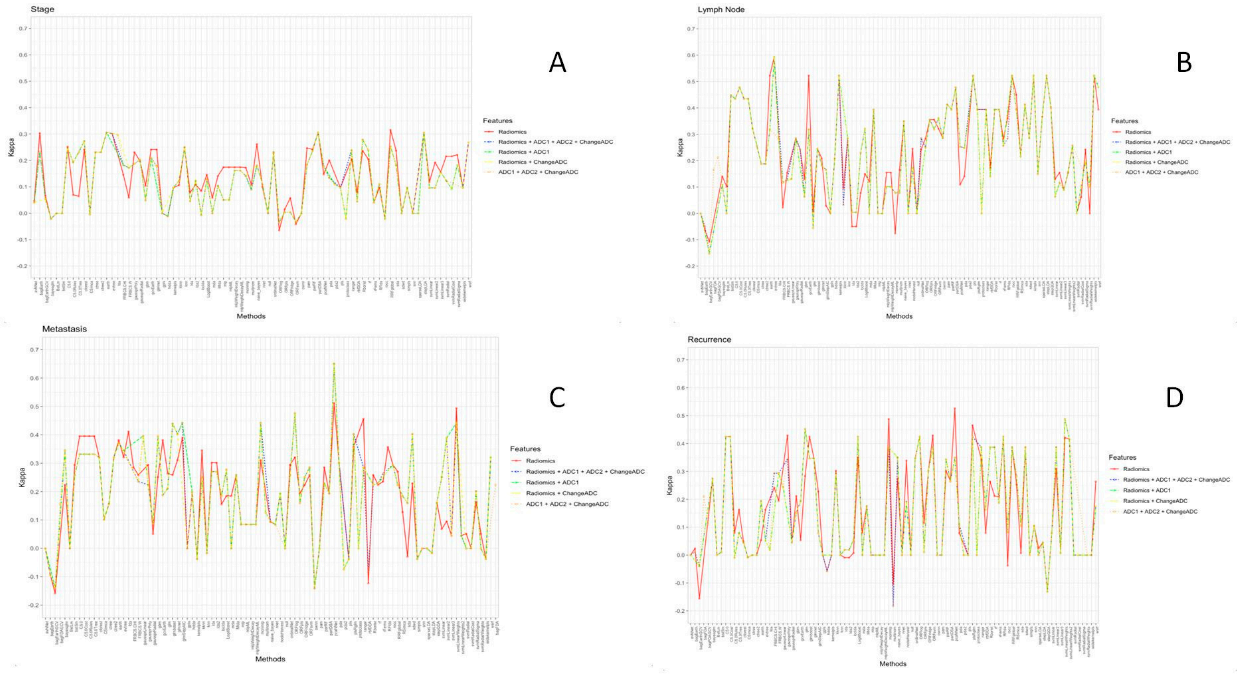

Figure S2. (**A**–**D**) Line plot is used to represent the variation in the efficiency metrics of the model using different sets of features and modeling algorithms. The above figure shows our various classifiers used to predict clinical outcomes with Kappa plotted on a scale of 0–1.

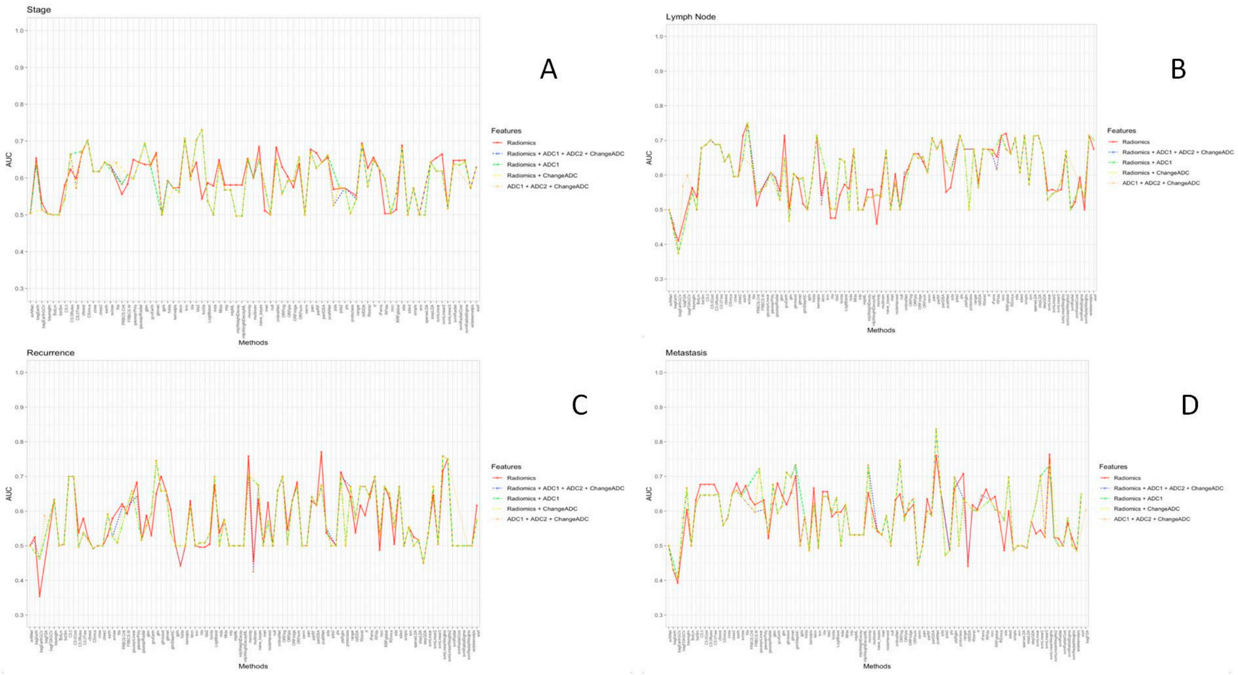

Figure S3. (**A–D**) Line plot is used to represent the variation in the efficiency metrics of the model using different sets of features and modeling algorithms. The above figure shows our various classifiers used to predict clinical outcomes with AUC plotted on a scale of 0–1.

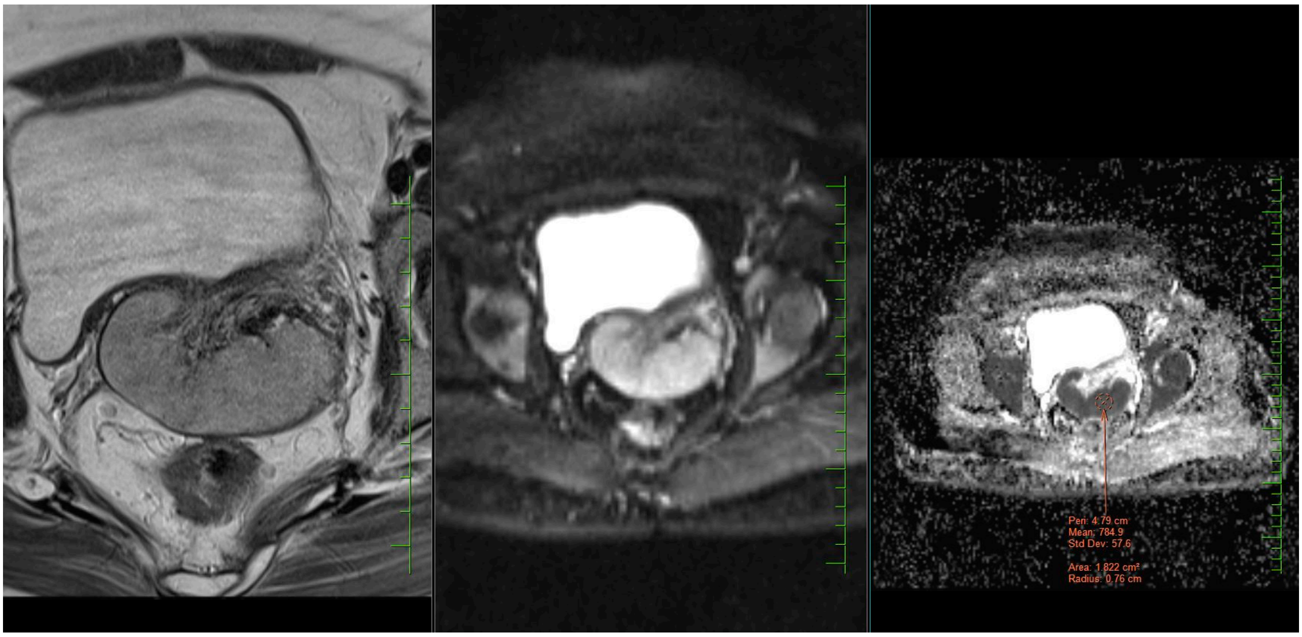

Figure S4. T2 WI and diffusion imaging showing representative method of ADC calculation.

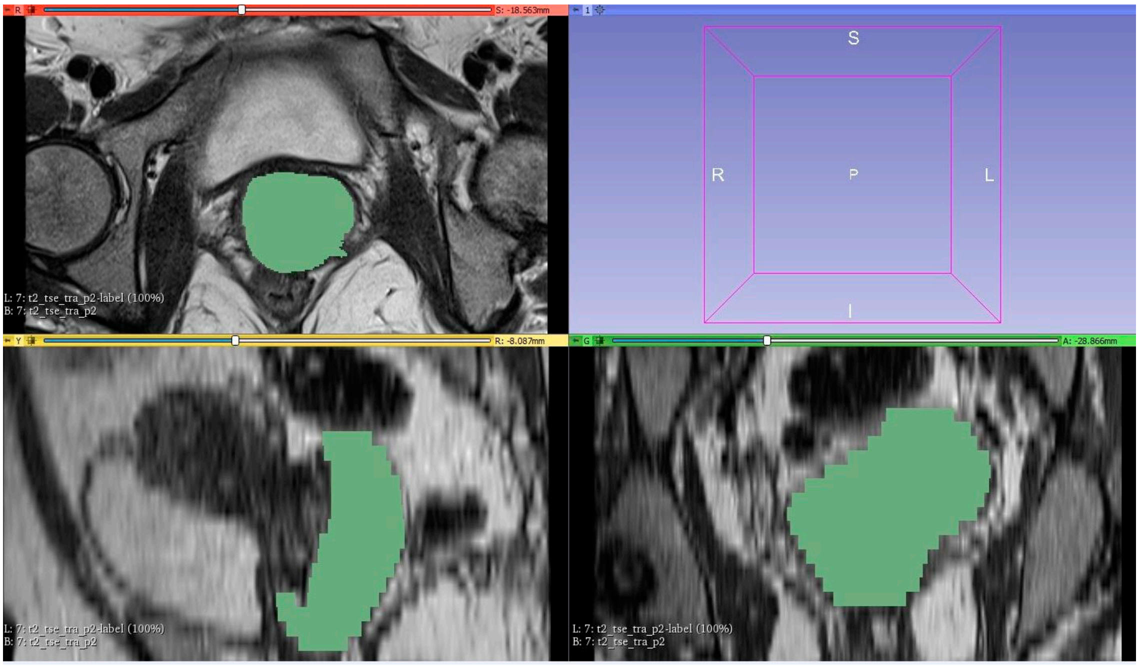

Figure S5. Segmentation process using Slicer-3d software and VOI delineation for radiomics feature extraction.
